# Virtual faculty development program in bioethics evaluated by Kirkpatrick model: A unique opportunity

**DOI:** 10.1371/journal.pone.0293008

**Published:** 2023-10-30

**Authors:** Tara M. A. Shallal, Nazdar Ezzaddin Alkhateeb, Ali Al-Dabbagh

**Affiliations:** Department of Medical Education, College of Medicine, Hawler Medical University, Erbil, Iraq; University of Sharjah College of Health Sciences, UNITED ARAB EMIRATES

## Abstract

**Background:**

With a shortage of teachers willing to deliver bioethics courses, particularly during the COVID-19 pandemic, faculty development in bioethics has become increasingly important for capacity building in medical education. This study aims to determine the impact of an online course on faculty development in teaching bioethics.

**Methods:**

This quasi-experimental study involved twenty-six multidisciplinary faculty members who participated in an online bioethics course from January 4th to 22nd, 2022. Kirkpatrick’s evaluation model was used to assess the participants’ reactions, knowledge, and behavior, using online questionnaires and direct observation by expert faculty. SPSS 25 was used for data analysis.

**Results:**

The Kirkpatrick evaluation model showed that the course was highly satisfactory in terms of content and delivery, with a significant improvement in clinical faculty’s knowledge from pre-test to post-test (14.18 ± 1.601 to 16 ± 2.049, p < 0.05). The participants demonstrated an improved range of teaching and learning methods.

**Conclusion:**

The online course on bioethics successfully improved the clinical faculty’s knowledge and overall approach to teaching bioethics. These findings highlight the importance of well-constructed faculty development programs, particularly during times of resource constraint such as the COVID-19 pandemic.

## Introduction

It has been shown that ethical issues often cast a shadow on health care in relation to organizational culture and the quality of services provided to patients [1]. In response to the wide acknowledgment of the range of ethical and legal issues that medical practice evokes, the World Medical Association recommended teaching medical ethics and human rights [2].

Faculty development of educators motivated to facilitate learning improvement on bioethics was found to contribute to the effectiveness of the bioethics curriculum [3, 4]. Learners therefore must receive a well-constructed program that addresses those issues and tackles them effectively through using appropriate methodologies as well as content [1]. Such competencies require special faculty development programs provided by universities. However, considering the adaptations that COVID-19 pandemic forced on medical practices as well as educational interventions, a substantial change to the virtual approach of training is needed to sustain faculty development programs [5, 6].

The essence of running all sorts of faculty development programs is to successfully implement them and achieve their goals. However, there remains a need to evaluate their impact in order to justify the time, and effort that are required to run these programs. The models of evaluation utilize critical appraisal of such programs, such as the Kirkpatrick’s, and the Moore’s framework of expanded outcomes. Choosing the appropriate model is especially important in resource-constrained settings available to a developing country like Iraq. Over the past decades, the country has suffered multiple blows to its medical education system [7–9].

The Kirkpatrick evaluation model is made of four levels that are reactions, knowledge gain, behavior, and impact. Each of these levels is required to be assessed when the resources are available for programs. It is indisputable that the Kirkpatrick model has made a significant contribution to evaluation theory and practice by providing a simple system for discussing outcomes and the way to obtain information on these outcomes. The Kirkpatrick model has gained recognition in a variety of evaluation studies as a result of these advantages [10].

Bioethics was introduced to the new integrated curriculum of Hawler Medical University in 2017. The need for capacity building in teaching bioethics led to establishing a bioethics unit as the Iraq-Erbil Unit International Chair in Bioethics, formerly UNESCO Chair in Bioethics (ICB).

This study aims to use Kirkpatrick’s evaluation model to determine the impact of an online course offered by the ICB on “faculty development in bioethics”.

## Methods

### Research design

A prospective quasi-experimental study was conducted, during the period from 4^th^ to 22^nd^ of January 2022, at Hawler Medical University. In this work, the impact of the course was evaluated at three levels using the Kirkpatrick evaluation model.

#### Recruitment of participants

To make sure participants would be fairly represented in the training, a notification was shared with the deans, in December 2021, to solicit nominations from each of the 5 colleges in the university. Hawler Medical University is the oldest medical university in Iraq. It comprises the colleges of Medicine, Dentistry, Pharmacy, Nursing, and Health Sciences. Twenty-six participants were selected by purposive sampling technique to take part in the training.

Upon confirming their participation, they were enrolled in a WhatsApp group for communication and the distribution of the course materials. All participants in in the course were asked to take part in the study. Sixteen members from the medical, nursing, and dental colleges, were clinicians, while the rest came from the basic science departments.

#### The intervention

This study was conducted for the evaluation of the new Training opportunity to Teach, Train and Transfer International Bioethics in Health Sciences course (3-T IBHSc). This course was organized by the international network of the ICB for medical teaching faculties at universities in Malaysia, Indonesia, and Sri Lanka and delivered as a multisession workshop.

It should be emphasized that the workshop was made available for the first time through online platform, due to COVID-19, as it was presented to participants from both Amrita Institute of Medical Sciences/India and Hawler Medical University/Iraq. Only the data of Hawler Medical University were used for the purpose of this study, with the identity of participants kept unknown to the researchers throughout the training.

The five online workshop sessions were distributed over five days with an overall 26 contact hours (around 5–6 hours/session). The sessions were carried out in a manner allowing multi-disciplinary teams of participants to train together effectively.

The goals of the workshop were to introduce participants to the current core curriculum, reflecting the Universal Declaration of Bioethics and Human Rights (UDBHR). In line with these goals, it was intended to enhance their knowledge and level of application of the principles of Bioethics, and provide them with the essential skills to use innovative methodologies for teaching and implementing formative and summative assessment in bioethics education.

The workshop sessions were facilitated by seven senior training faculties from the Education Department of the ICB. The training course used the methodology of a group of “co-learner” concept, where training faculty and participants are co-learners, using the process of “learning through osmosis” [11]. This is an analogy for the indirect way of learning that takes place while workshopping through role-playing, debates, and short videos. The participants were assembled as a single unit for certain online sessions, and they met up face to face as segregated teams for group activities. There were sixteen thematic topics selected for presentation by the participants. The main course coordinator (co-chair) designed the program schedule based on the UNESCO Bioethics Core Curriculum and “14 Ethics Teachers’ Training Courses”. For this workshop, Hawler Medical University selected 26 participants.

There were 5 workshop sessions; in each session, there were four topics of bioethics, and multiple activities related to them. The fifth session was distinguished from the others with the inclusion of a simulated teaching assessment of participants’ application of delivery methodology in bioethics teaching.

In depth discussions of the issues, during plenary sessions, were ensured by the facilitators, who would respond to the co-learners’ inputs. The training emphasized the importance and universality of the bioethics principles as described in the Universal Declaration on Bioethics and Human Rights. In addition, the training faculty emphasized the importance of conveying the role of local application of these principles by participants.

#### Training evaluation

Using the Kirkpatrick evaluation model’s first three levels, evaluation forms of the effectiveness of this training course were sent by the training faculty in Department of Education in ICB.

The feedback of the participants for level I was taken at the end of the training workshop. It included a number of requirements such as their reaction to the content, relevance, agreement with objectives, interaction, range of topics covered, organization, time allotment and the overall arrangement [12]. The requirements were projected into the feedback questionnaire through ten questions. The first two questions were on rating the conduct of the workshop as per content and relevance (scale of 1–5; 1 not very satisfied and 5 very much satisfied) for its conduct. The other eight questions were rated by Likert Scale 1–5 (5 = strongly agree, 4 = agree, 3 = neutral, 2 = disagree, 1 = strongly disagree), (See the S1 File: Course feedback questionnaire).

Level II data were analyzed and presented to reflect two different time periods: before and after the workshop sessions. Before the beginning of the training course, participants were required to answer a questionnaire distributed online. The questionnaire was composed of two parts, demographic information and a pretest. The pretest, on a semi-structured questionnaire was designed to record the participant’s knowledge and consisted of twenty-five questions (See S2 File: Pre- and post-test questions). To address the different learning levels of Blooms taxonomy, the questionnaire included twenty items that tested knowledge and understanding of the core bioethics. The other five were case based scenarios which tested the level of applying ethical decisions and resolving ethical dilemmas. A post-test with participant’s written responses to the course was obtained at the conclusion of the training. A post-test with participant’s written responses to the course was obtained at the conclusion of the course. In the tests the score was one and zero for a correct and incorrect response respectively.

Level III using Kirkpatrick model, tested if the impact of learning has been transferred to the participants’ methods of teaching. This was done by observing the implementation of the new methods they learned in the assessment session. The process was administered by the trainer faculty from the Department of Education of the ICB. The participants were divided according to their specialties, to observe their choice of method of teaching online as a change in their ‘behavior’ towards teaching bioethical issues.

#### Data analysis

For all levels of Kirkpatrick model, the questionnaires were collated, coded, and the obtained data were analyzed by software package Social Science SPSS 25.

Both percentages and the mean scores±standard deviation (SD) were used to illustrate the perceptions and reactions as well as the responses to the tested bioethics knowledge. We compared the mean score values of the participants for each component of the knowledge variable before and after the course using a paired t-test (participants who did not take both pre- and post-test were not included). A *P* value less than 0.05 was regarded as significant.

Ethical approval was obtained for this study as part of a PhD project from the Ethics Committee in the College of Medicine in Hawler Medical University under the number 7/1 issued on 12/07/2021, and the written consent of participants was taken.

## Results

An interventional study was conducted on 26 participants from Hawler Medical University. 17 (65.3%) of them were females, while 9 (34.6%) were males.

Eighteen participants took the pretest. However, 16 (88.9%) of them completed the post-test questionnaire. There were four participants of the 26 who completed the post-test, without taking the pre-test.

**Fig 1:**
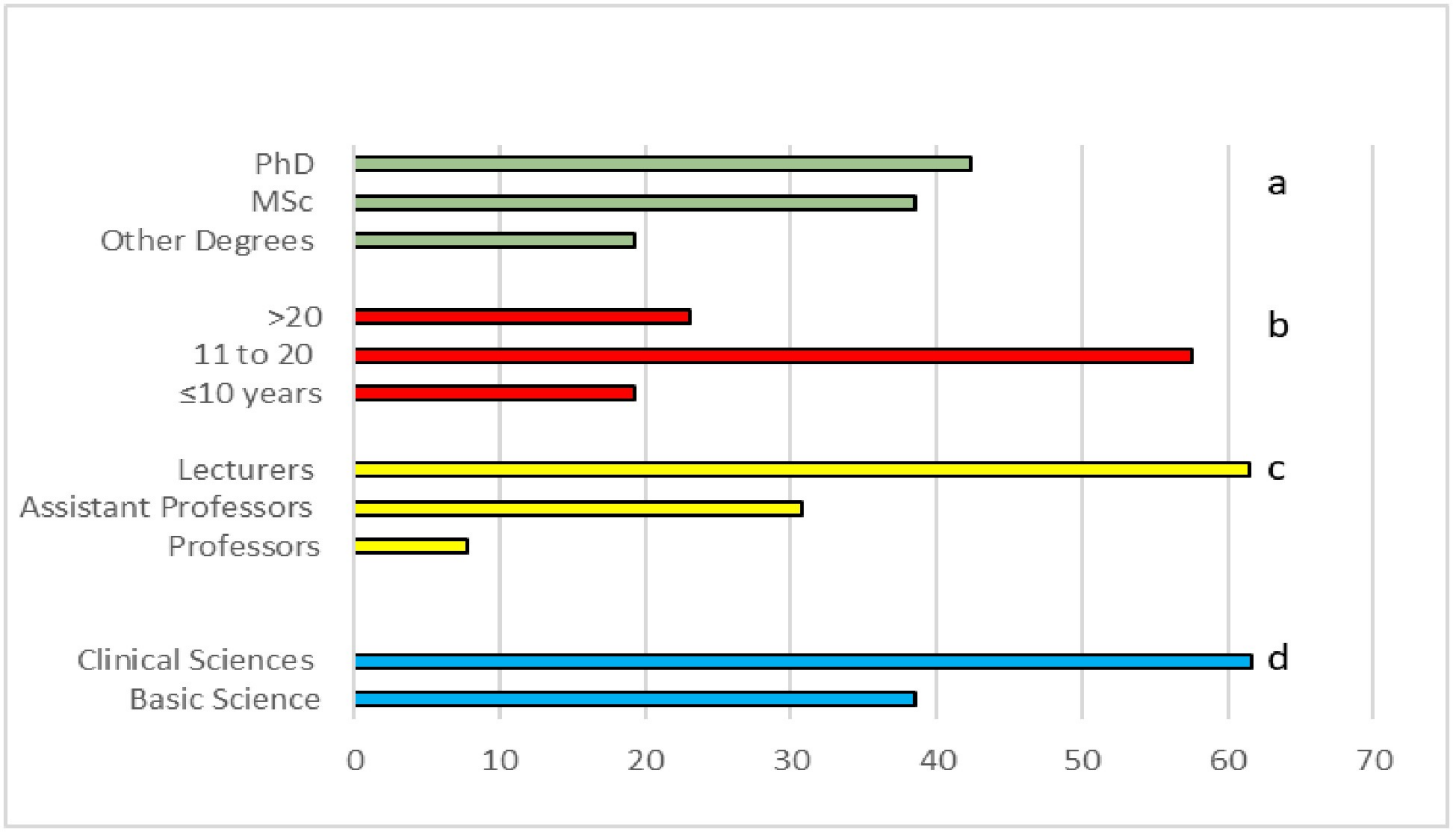
Demographic information of the participants in percentage (%) a academic degrees, b years of teaching experience, c academic titles, and d academic affiliation.

The response rate 69.2% (18 of the 26). Fig 1 contains information on the demographics of participants.

The outcome of the Kirkpatrick evaluation model level I showed that out of the 18 participants who answered the feedback questionnaire, 12 participants (66.7%) were very satisfied with the course content and the overall conduct. Fifteen participants (83.3%) thought that it was very relevant to their practice as healthcare professionals. The scope and different results obtained from the questionnaire are listed in Table 1.

**Table 1:** Participants’ perceptions survey results (n = 18).

Item	N (%)	Mean±SD
**1**. Satisfaction with course content and overall conduct	12(66.7)	4.61±0.607
**2**. Relevance of the conducted course	15(83.3)	4.83±0.383
**3**. Objectives were clear	10(55.6)	4.11±1.323
**4**. Participation and interaction were encouraged	10(55.6)	4.05±1.392
**5**. The topics covered were relevant to the training objectives	11(61.1)	4.22±1.308
**6**. The content was organized and easy to follow	10(55.6)	4.11±1.278
**7**. The training will help me to integrate bioethics into my teaching	11(61.1)	4.22±1.262
**8**. The faculty was adequately prepared and knowledgeable	10(55.6)	4.11±1.367
**9**. The time allotted for the course was sufficient	6(33.3)	3.83±1.098
**10**. The overall arrangements were adequate and comfortable	8(44.4)	4.05±1.211

**Fig 2:**
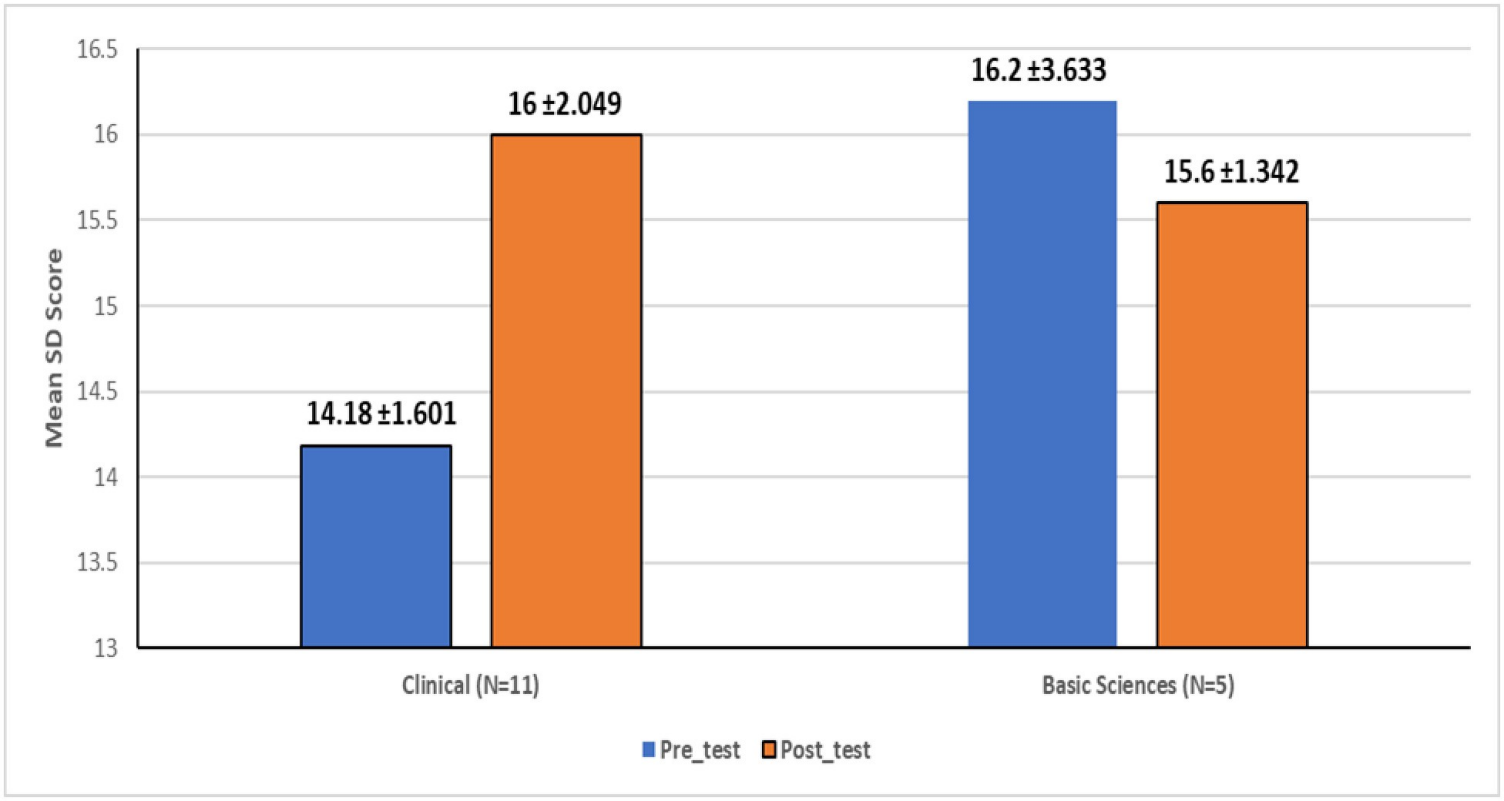
Mean pre- and post-test (level II Kirkpatrick model).

The results of level II of Kirkpatrick evaluation model revealed a significant difference (p < 0.05) between the mean test results before (pretest) and after (post-test) the intervention among the participants from clinical departments (n = 11(68.8%)). There was no significant difference in the learning gain for those participants from the basic science departments (n = 5(31.3%)) as shown in Fig 2.

Regarding the Kirkpatrick evaluation model level III, the choice of method of teaching was observed. Out of the 16 participants who attended the last workshop session, 10 (62.5%) used case scenarios, 3(18.8%) used photography, 2(12.5%) went for using videos, and only 1 participant (6.3%) resided to role play to deliver teaching of the ethical issues.

## Discussion

As a program evaluation model, Kirkpatrick has become the one that is most widely known [10]. Thus, it was used when a new faculty training was carried out with the intention of enhancing teachers’ professional capacities for instructing students in bioethics. The ultimate aim was to assist students in learning and practicing the principles of bioethics. The 3T-IBHSc is now a training being used in a number of programs across the globe to introduce the new concept to faculty members and assess their performance [6, 13]. Kirkpatrick model showed that the participants’ perceptions of the workshop were noteworthy regarding course content, relevance, and the overall conduct. The findings of this study are consistent with the findings of Ramalingam and Bhuvaneswari [14].

Our analysis showed that 3T IBHSc improved the performance of the participants in an online situation. At the end of the training course, the knowledge gained by the participants from clinical departments was significant. These findings are in agreement with other studies reporting similar results [15–17]. Here it is worth mentioning that the overall increase in the knowledge of participants from basic science departments, did not match that of the participants from the clinical departments. This might be explained by the fact that the participants numbers from basic science departments were much less than the number of those from clinical departments. It can also be explained by the notion that clinicians face a larger range of bioethical issues, more commonly and regularly [18]. Although other studies [14] report higher score results on level two Kirkpatrick, those studies are based on in-person training processes, while in our case we used the online model.

Despite the uniqueness of such a course that has been in demand by our staff, the online delivery of faculty development programs is still at an infantile stage in our country in terms of feasibility and effectiveness. This is probably due to a general lack of participants’ experience with online platforms that provide most of the setting for the training course as well as its evaluation. This is when some studies still report the preference for on-site training to enhance participants performance and satisfaction [19].

Evaluating behavioral changes, showed that the majority of clinicians chose case vignette as the preferred method of teaching. This observation that can be partly explained by the fact that the majority of clinicians are familiar with the case-based learning as the method of choice for theoretical and practical teaching [18]. Additionally, there was limited use of other recommended methodologies of teaching that are known to stimulate audience to reflect and discuss the underlying bioethical issues. Such limitations are noted in other studies reporting training in a virtual medium [20–22].

The limitations of this study are that some of the evaluations were based on participant’s perceptions, which are a potential source of bias for the capacity development results. Furthermore, the number of participants allowed by the organizers was narrowed to 26, so the generalizability of our findings is affected. We therefore, recommend applying a mixed method approach in the future, by using semi-structured interviews in qualitative research. And involving other stakeholders to provide feedback such as administration, and students.

## Conclusions

Online faculty development programs in bioethics are effective tools for capacity building and are highly satisfactory in improving clinical faculty’s knowledge of bioethics. Case-based learning emerged as the preferred teaching method. Kirkpatrick’s evaluation model proved useful in evaluating the effectiveness of such programs, especially in resource-limited settings. These findings underscore the importance of well-designed and evaluated faculty development programs in medical education, globally, to improve the quality of healthcare services delivered to patients.

## Supporting information

S1 File (PDF)

S2 File (DOCX)

S3 File (XLSX)

## Abbreviations

ICB: International Chair in Bioethics
3-T IBHSc: Teach, Train and Transfer International Bioethics in Health Sciences course
UDBHR: Universal Declaration of Bioethics and Human Rights
