# Supplementary material for: Virtual faculty development program in bioethics evaluated by Kirkpatrick model: A unique opportunity
Source: PLoS One. 2023 Oct 30;18(10):e0293008. doi: 10.1371/journal.pone.0293008 (PMC10615268; doi:10.1371/journal.pone.0293008)
Supplement: S1 File — (PDF) [file pone.0293008.s001.pdf]

3T- IBHSc International Course In Bioethics for Healthcare Professionals Hawler Medical University, Erbil, Iraq: Overall Course Feedback

This Course Feedback Form is intended to record your reactions to the training that you have just completed. It is not intended as an evaluation tool. Your feedback is vital to us for a number of reasons:

- Your feedback will assist us in understanding effectiveness of the training and /or special circumstances that may have influenced this particular workshop/ training.
- The feedback helps us to better plan our transfer of learning (TOL) activities following training.
- Your response will provide information about the use of training methodologies and will supply suggestions to further improve future programs.
- Your responses will assist us in providing support and better development of trainer faculty.
- Your responses will assist us in identifying barriers to curriculum/future training programs.

Thank you for your thoughtful appraisal and for your commitment to providing quality training in Bioethics.

* Indicates required question

1. How satisfied were you with the course content and overall conduct of the Course? *

Mark only one oval.

	Not very
1	⬭
2	⬭
3	⬭
4	⬭
5	⬭
	Very much

2. Was the training conducted relevant and helpful for your job as a healthcare faculty / professional? *

Mark only one oval.

	Not very
1	◯
2	◯
3	◯
4	◯
5	◯
	Very much

3. What were overall highlights of the Course for you? *

____

____

____

____

____

4. What were the areas that could be improved in the overall conduct of the Course? *

5. Please indicate your level of agreement with the statements listed below *

Mark only one oval per row.

	Strongly agree	Agree	Neutral	Disagree	Strongly disagree
**The course objectives were clearly defined and met by the end**	⬭	⬭	⬭	⬭	⬭
**Participation and interaction were encouraged**	⬭	⬭	⬭	⬭	⬭
**The topics covered were relevant to the training objectives**	⬭	⬭	⬭	⬭	⬭
**The content was organized and easy to follow**	⬭	⬭	⬭	⬭	⬭
**The training will help me to integrate bioethics into my teaching**	⬭	⬭	⬭	⬭	⬭
**The faculty was adequately prepared and knowledgeable**	⬭	⬭	⬭	⬭	⬭
**The time allotted for the course was sufficient**	⬭	⬭	⬭	⬭	⬭
**The overall arrangements** were adequate	⬭	⬭	⬭	⬭	⬭

were adequate and comfortable

6. The permission for research: *

Mark only one oval.

- ( ) I give my permission to use this information for research purposes
- ( ) I don't give my permission to use this information for research purposes

This content is neither created nor endorsed by Google.

Google Forms
