# Supplementary material for: Virtual faculty development program in bioethics evaluated by Kirkpatrick model: A unique opportunity
Source: PLoS One. 2023 Oct 30;18(10):e0293008. doi: 10.1371/journal.pone.0293008 (PMC10615268; doi:10.1371/journal.pone.0293008)
Supplement: S2 File — (DOCX) [file pone.0293008.s002.docx]

**Pre/post- test questions**

**1. What do we mean by ethics?**

1. Moral judgments
2. Determinants of what is right or wrong
3. Rules or standards governing a profession
4. Elements of all of the above

**2. What are Human Rights?**

1. Special privileges awarded to criminals and prisoners
2. What the European Parliament decides is right
3. Rights listed in the UN Declaration of Human Rights 1948
4. Rules designed to frustrate legal proceedings

**3. Which of the following is *not* a recognized form of organizational justice?**

1. Procedural justice
2. Interactional justice
3. Shareholder justice
4. Distributive justice

**4. Autonomy is one of the main principles of bioethics , which mean:**

a. Selfishness

b. Self-awareness

c. Self-promotion

**d.** Self-governance

**5. Which of the following ethical issues form the foremost part of Hippocratic Oath?**

a. Confidentiality

b. Sexual boundaries

c. Advertising

d. Bribery

**6. Paternalism among doctors is not an ethical attitude because it conflicts with**

a. Patient’s medical care

b. Doctors’ tasks and duties

c. Patient’s autonomy

d. Doctor’s autonomy

**7. The concept of justice in ethics is:**

a. an obligation of the patient to the society.

b. that the health resources must be distributed according to the principals of equity.

c. taken as patients right to choose or refuse treatment.

d. For all medical Professionals to do good for all patients under circumstances

**8. Confidentiality can be breached**

a. In the case foreign nationals in a country.

b. When financial resources are scarce and patient is not compliant.

c. When a patient authorizes to do so

d. For a patient who requires Invasive treatment

**9. Primum Non Nocere means**

a. First, do no harm

b. First, do not listen

c. Never be the first

d. The higher, the fewer

**10. According to the ethical principles, the benefits we are obliged to provide as healthcare professionals are specified in part by ...**

a. Our upbringing and personal values

b. Our relationship, role, and agreements

c. Our employer, the law, our conscience

d. Our contract with the hospital or clinic

**11. The physician should do what is medically indicated, do good than possible harm .” That is called the:**

a. Hippocratic oath

b. Medical Nonmaleficence Principle

c. Medical Indications Principle

d. Best Interests Principle

**12. Medical Ethics:**

a. Is the study of moral aspects of a doctor’s professional life?

b Is regulated by local Medical and Dental council

c. Is the code of conduct of doctor’s professional life?

d. Is covered by Hippocratic Oath.

**13. The principles of medical ethics are all, except:**

a. Non-Maleficence

b. Beneficence

c. Autonomy

d. Confidentiality

**14. Identify the most influential event that led to the HHS Policy for Protection of Human Research Subjects:**

A. Nuremberg trials

B. Syphilis Study at Tuskegee

C. Jewish Chronic Disease Hospital Study

D. Willowbrook Study

**15. The Belmont Report is significant because:**

A. It was written by the National Commission for the Protection of Human Subjects.

B. It articulated ethical principles that formed the basis for the HHS Human Subjects Regulations.

C. Belmont is another word for individual autonomy and respect.

D. It was a seminal document about the concept of informed consent.

16. In order to participate in research, children must:

A. Provide written informed consent

B. Provide written permission

C. Provide assent, unless the IRB determines that they are too young

D. There is no need of ascent or informed consent for children

17. For research involving pregnant women, participation requires:

A. That women have completed the first trimester.

B. That the study be conducted first in men.

C. Permission of the father.

D. Consideration of risks and potential benefits for the fetus and the pregnant woman.

18. The three fundamental principles of Informed consent are:

A. Voluntariness, Equipoise, Respect

B. Voluntariness, Comprehension, Disclosure

C. Benefits, Comprehension, Privacy

D. Disclosure, Equipoise, Privacy

19. Which of the following are not necessary for a proper medical record?

a. Timely and legible entries

b. Accurate and complete entries

c. Corrections made by blacking out error

d. A logical progression of entries with reference to concurrent records and former entries

20. Informed consent is not required when

a. the procedure is simple and common.

b. there is a life-threatening emergency.

c. the patient’s mental status prevents a reasonable informed consent.

d. All of the above.

# Case study 1: A 25 years old healthy male dies in a fatal road traffic accident; he has advance directives about his organ donations. Health care rationing of organs take multiple factors into account for deciding of who will be recipient of organ. Social utility, equity, equality, distributive justice, beneficence and expected life expectancy after organ transplantation ,all perspectives play roles in allocating of scarce organs to recipients, as waiting lists are lengthy and decision is tough.

# The most likely recipient of organ will be:

# A 45 years alcoholic with irreversible liver failure.

# A 75 years old female with extreme form of dementia and end stage renal failure.

# A 35 years young entrepreneur with acute renal failure.

# A 10 years old male with multi organ failure and brain death.

# A 40 years old female with heart failure and HIV positive.

# Case study 2: a 34 years old female, comes to emergency reception (ER) with her 20 days old baby boy, suffering a lesion in his groin. Mother tells that her baby isnot his usual active. Baby has Mycoscoriasis fungal infection of neonates. ER physician on physical examination finds baby alert, pink and active; sends him back to home. Although Nurse in ER had noticed the lesion in groin and suspects that her previous experience is that such lesions in neonates, if not treated timely, led to fatal outcomes. Baby returns in ER after 24 hours with many lesions on his trunk, severe breathlessness, cyanosed and unconscious.

# Health care providers were liable to commit the form of harm to the patient:

# Advertent negligence

# Assault

# Battery

# Inadvertent negligence

# Malpractice

# Case study 3: A 35 years old male, athlete admits with pain in Right knee, investigations reveal that he is suffering from end stage osteosarcoma, and needs extensive chemotherapy, radiotherapy to treat the cancer. Athlete’s father died with osteosarcoma in his fifties. His mother died due to breast cancer in her forties. He has a living will that if he ever diagnosed with cancer, not wanted to be aggressively treated by chemotherapy, radio therapy and other therapies. Physician is consistent to treat him with all sorts of treatment possible to cure his cancer.

# Physician is exercising:

# Act of Paternalism

# Ideal beneficence

# Obligatory beneficence

# Specific beneficence

# Surrogate decision maker

# Case study 4: A 2 days old baby girl is admitted in neonatal intensive care unit; she is suffering from early onset neonatal sepsis accompanied with multi organ failure. She has to put on mechanical ventilatorsupport; her respiratory status is moderately working, pupils are fixed dilated, motor and sensory responses are absent and herheart is having regular sinus rhythm. Physician counsels parents, about brain death;and explains that it is of no use to keep the baby alive with evident brain death, and asks for parents’ permission to off the artificial mechanical support.But, Parents are stubborn to continue life support.

# Social and ethical principles come in conflict with health care system are:

# Cost related to futile treatment and medical concerns.

# Parents’ wishesand futile medical treatment

# Respect for autonomy of surrogate decision maker.

# Religious and cultural beliefs to not to end life.

# Virtue ethicsand ideal beneficence versus medical realities.

## Case study 5:A 30 years old pregnant woman with 37 weeks of gestation admits in maternity ward with complaints of starting labor pains, she is human immunodeficiency virus (HIV) positive. Her previous two children are HIV positive. She delivers healthy baby boy weighing 3.5Kg.

She is not willing to test her baby for HIV screening and left the ward against medical advice.

To save public from preventable HIV transmission, policies developed by health care policy makers must address the policy:

1. Compulsory and voluntary screening for HIV for all.
2. Mandatory HIV screening of all newborns.
3. Mandatory screening of all pregnant women for HIV.
4. Mandatory screening for HIV for all irrespective of low and high risk groups.
5. Voluntary selective screening for HIV, indulge in unsafe sexual practices.
